# PRES after spinal anesthesia

**DOI:** 10.1007/s10194-011-0335-3

**Published:** 2011-03-27

**Authors:** Satoru Takeuchi, Kimihiro Nagatani, Naoki Otani, Hiroshi Nawashiro

**Affiliations:** Department of Neurosurgery, National Defense Medical College, 3-2 Namiki, Tokorozawa, Saitama 359-8513 Japan

We read with interest the recent article by Pugliese et al. [1] concerning a case of intracranial hypotension and PRES and the debate that followed [2, 3]. The authors reported a rare patient who presented worsening of headache and seizures at 7 days after spinal anesthesia. Brain MRI showed signs of intracranial hypotension (IH) and posterior reversible encephalopathy syndrome (PRES). Therefore, the authors diagnosed the patient as PRES caused by IH, secondary to spinal anesthesia. We wish to provide further comment. Reversible cerebral vasoconstriction syndrome (RCVS) is characterized by severe acute headache and constriction of cerebral arteries [4, 6]. IH is also a cause of RCVS [5, 7]. RCVS should not be confused with PRES although there is overlap in both the presumed underlying mechanisms and the predisposing factors [5]. Indeed, some patients can actually exhibit RCVS with PRES [4, 5]. The authors ruled out RCVS, mainly because of the subacute and progressive onset of the clinical symptoms and normal MRA findings. However, it is possible that the characteristics of headache may be changed by anesthesia. Further, Ducros et al. [6] described that, in some patients who had an initial normal MRA at a mean of 5.5 days (range 2–9 days) after headache onset, a repeat MRA showed visible narrowings at a mean of 13.6 days (range 9–20 days). In contrast, a repeat MRA was not performed in the cases presented by Pugliese et al., suggesting that the authors cannot rule out the possibility of RCVS.
